# Supplementary material for: Getting More Out of Biomedical Documents with GATE's Full Lifecycle Open Source Text Analytics
Source: PLoS Comput Biol. 2013 Feb 7;9(2):e1002854. doi: 10.1371/journal.pcbi.1002854 (PMC3567135; doi:10.1371/journal.pcbi.1002854)
Supplement: Dataset S1 — GATE software. Dataset S1 bundled with this paper contains a distribution of GATE (or see http://gate.ac.uk/download/). (TGZ) [file pcbi.1002854.s001.tgz › plos-gate/src/gate/creole/annic/apache/lucene/analysis/standard/package.html]

A grammar-based tokenizer constructed with JavaCC.

Note that JavaCC defines lots of public classes, methods and fields
that do not need to be public.  These clutter the documentation. 
Sorry.

Note that because JavaCC defines a class named Token, org.apache.lucene.analysis.Token
must always be fully qualified in source code in this package.
